# The Use of Image Analysis to Detect Seed Contamination—A Case Study of Triticale

**DOI:** 10.3390/s21010151

**Published:** 2020-12-29

**Authors:** Łukasz Gierz, Krzysztof Przybył, Krzysztof Koszela, Adamina Duda, Witold Ostrowicz

**Affiliations:** 1 Institute of Machine Design, Faculty of Mechanical Engineering, Poznań University of Technology, Piotrowo 3, 60-965 Poznan, Poland; lukasz.gierz@put.poznan.pl (Ł.G.); ostrowicz.witold@gmail.com (W.O.); 2 Department of Food Technology of Plant Origin, Faculty of Food Sciences and Nutrition, Poznań University of Life Sciences, Wojska Polskiego 31, 60-624 Poznan, Poland; krzysztof.przybyl@up.poznan.pl (K.P.); adaminaduda@wp.pl (A.D.); 3 Department of Biosystems Engineering, Poznan University of Life Sciences, Wojska Polskiego 50, 60-625 Poznan, Poland

**Keywords:** triticale, entropy, image analysis and processing, artificial neural networks

## Abstract

Samples of triticale seeds of various qualities were assessed in the study. The seeds were obtained during experiments, reflecting the actual sowing conditions. The experiments were conducted on an original test facility designed by the authors of this study. The speed of the air (15, 20, 25 m/s) transporting seeds in the pneumatic conduit was adjusted to sowing. The resulting graphic database enabled the distinction of six classes of seeds according to their quality and sowing speed. The database was prepared to build training, validation and test sets. The neural model generation process was based on multi-layer perceptron networks (MLPN) and statistical (machine training). When the MLPN was used to identify contaminants in seeds sown at a speed of 15 m/s, the lowest RMS error of 0.052 was noted, whereas the classification correctness coefficient amounted to 0.99.

## 1. Introduction

Sowing is one of the most important operations in the cultivation of cereals and other crops, because it determines the success of all subsequent operations and the essence of agriculture, which is specific and the most important branch of economy for human survival. Currently, machines with a very large working width are increasingly used for sowing. They work independently or are used as components for cultivation and sowing units. They seem to indicate the dynamic development of simplified sowing methods [1,2,3]. These are usually machines with a mechanical central seed metering unit (sowing unit) and pneumatic transport and distribution of seeds into individual rows [4]. Seeds are separated in special distribution heads invented by Weist in the 1950s [5]. The advantage of this system is its lightness and simplicity. The seed drill can be easily folded for road transport. While pneumatic seed drills have a lot of advantages, such as high efficiency, the possibility to be mounted on a combined cultivator, quick and easy loading of seeds, they also have some disadvantages. One of the most important disadvantages is low lateral sowing evenness, especially in hilly terrains [6,7].

There are a lot of scientific studies on the causes of uneven sowing of seeds with pneumatic seed drills [8,9,10,11,12,13] and methods of assessment of sowing evenness [14,15]. Researchers conducted experiments on the numerical simulation of seed dosing [16] and the separation of the mixture of airstream and seeds in the distributor head of the seed drill [17,18,19]. Unfortunately, these authors did not conduct any research to find out how seed contamination influenced the evenness of the distribution of the dosed stream.

It is usually impossible to control sowing in individual seed ducts [20] and to detect impurities or damaged seeds in commonly used universal and pneumatic seed drills. There have been scientific reports with examples of attempts to solve this problem with optical sensors [21]. There have also been experiments on piezoelectric sensors placed inside the distributor head of the seed drill [22] and tests of the sensitivity of various sensors [21,23]. Patent literature provides an example with an attempt to use capacitive sensors—US Patent 4782282 A. However, the experiment showed that this method was not promising due to slight changes in the capacitance of the sensors. Researchers from the University of Paderborn invented a honeycomb-shaped piezoelectric sensor, mounted on top of the distribution head and in the ducts, which can count seeds and monitor their flow [24]. For many years scientists have been working on various sowing control systems, mainly based on photoelectric sensors detecting individual seeds. One of the latest and heavily advertised achievements in this field is the ‘seed eye’ system developed by Väderstad [25].

A similar system was developed at the Poznań University of Technology between 2009 and 2012 [5]. It was also based on photoelectric sensors built from a single photoelement. This single photoelement was placed at the point of seed flow concentration on the outer arc of the measuring elbow. As a result, it was not necessary to X-ray the entire cross-section of the channel and thus the construction of the sensor could be simplified.

However, no seed quality control system has thus far been developed, i.e., a system detecting seed contamination as well as small and damaged seeds, which do not guarantee a good yield.

At present, there are attempts to find optimization solutions to effectively identify various decision-making processes in real time. This study is based on a method of artificial intelligence, i.e., artificial neural networks.

Recently, artificial neural networks (ANN) have been used to classify [26], analyze data [27], predict [28,29,30], control [31] and assess the quality of biomaterials [32,33]. What is an important practical feature of ANN is the fact that trained neural models work online. This enables continuous control (monitoring) of the identification of contamination of triticale seeds in real time.

Classification means finding a classifier that will enable the division of a set of elements into groups called classes. Elements belonging to one group are called objects [26,34]. They may differ from each other, but not in the properties due to which they are assigned to a particular class. The seeds in our research were divided into three classes of triticale: select, screenings and chaff.

Multilayer Perceptron (MLP) is an example of a neural network that is most often used in classification problems [35,36]. It is a unidirectional network, trained with the trainer method. It has multilayer architecture with at least one hidden layer. Only communication between neurons in neighbouring layers is possible. The activation function for hidden neurons is non-linear (sigmoidal). It is a unidirectional network, trained by a teacher, which has a multi-layer architecture with at least one hidden layer. The principle of operation of the unidirectional network is based on training, which uses a backpropagation algorithm. This means that the method enables the selection of modulating variables (weights) so that the data error (i.e., Root Mean Square Error) is as low as possible [30]. The set of modulating variables enables the acquisition of information from other neurons. The weights determine the values of the matched data by connecting nodes between individual neurons. The activation function for hidden neurons is non-linear (sigmoidal) [30,37,38].

The way of preparing the dataset is important in classification. Recently, image analysis has also played a major role in various research areas. Image analysis includes: segmentation [39], location of objects and the identification of geometric parameters [40], models of colors [41,42], detection of edges and texture from the image. The computer imaging process is not easy. It is complex and takes time to get a promising result [43]. Consequently, the existing methods of image analysis are continuously modernized.

The aim of this study was to apply one of the methods of artificial intelligence, i.e., neural networks, to identify contaminants in seeds by means of image analysis and a camera for displaying quick photos. The behavior of three main variants of triticale seeds sown by pneumatic seed drills was investigated: select triticale, triticale screenings, and triticale chaff.

## 2. Materials and Methods

### 2.1. Preparation of Samples

**Figure 1:**
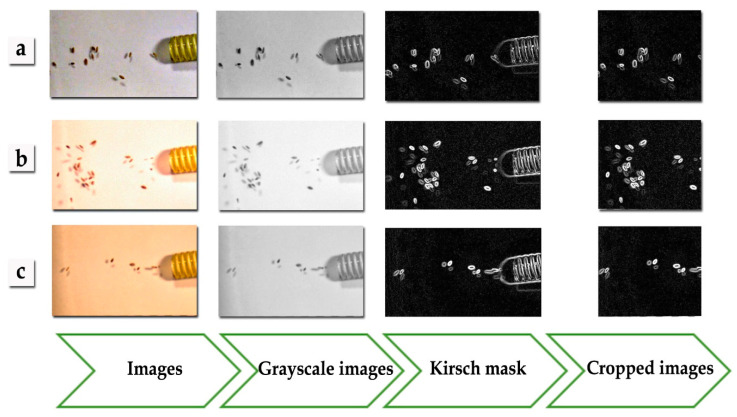
An example of creating a photo pattern for the select triticale class, with seed transport speeds of: (**a**) 15 m/s, (**b**) 20 m/s and (**c**) 25 m/s.

The most common cereal seeds sown in Poland were used in the study. The research material consisted of triticale seeds of the Rotondo cultivar, whose initial moisture content in each class of triticale was as follows (Figure 1): select—9.8%, screenings—10.7%, and chaff—9.9%. Triticale seeds were harvested on one of the local farms in the town of Kórnik, Wielkopolskie Voivodeship, Poland.

At the next stage of the research the dose of seeds sown was determined—it was based on the most common dose in Poland, i.e., 168 kg/ha. Before the experiments had an adequate stream of the mass of seeds flowing through the pneumatic conduit was selected, so that it would correspond to the dose of 168 kg/ha, i.e., the sowing density was 400 pcs/m^2^. The sowing density was calculated according to the following formula [44]:

(1) $$\mathrm{sowing}\;\mathrm{density}\;=\frac{\left(\mathrm{number}\;\mathrm{of}\;\mathrm{seeds}\;\mathrm{sown}\;\cdot \;\mathrm{germination}\;\mathrm{capacity}\right)}{\mathrm{TKW}}$$

where:

- sowing density. the number of plants per area unit (pcs/m^2^),
- number of seeds sown (kg/ha),
- TKW—Thousand Kernel Weight (g), and
- germination capacity—for qualified seeds MINIMUM 80% for triticale.

### 2.2. Test Facility

**Figure 2:**
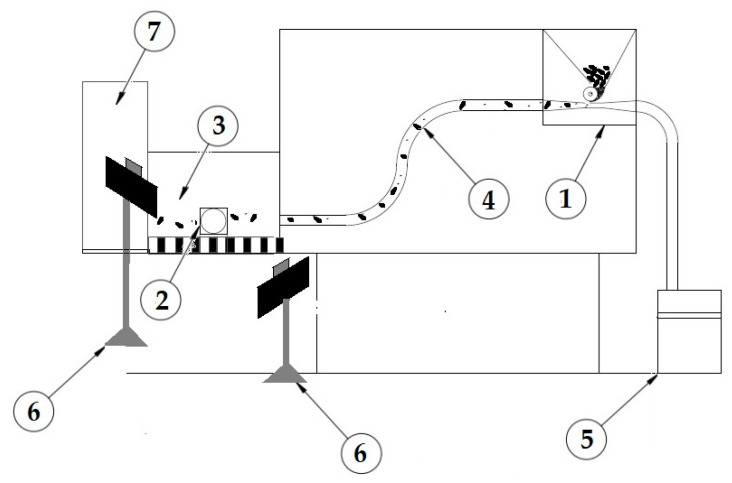
A scheme of the test facility, 1—drilling unit (a feeder), 2—camera, 3—screen with a scale, 4—pneumatic hose, 5—vacuum cleaner with the blower function, 6—LED lighting with a stabiliser, and 7—seed container.

An original test facility was built for the experiment in order to reflect the actual operating conditions of the sowing control system as much as possible. The facility consisted of the following elements (Figure 2):

- a seed sowing unit, including: a seed dispenser and sowing rollers driven by an electric motor with adjustable rotational speed of the pneumatic channel (1),
- a Chronos 1.4 camera (2),
- a screen with a scale (10 mm) (3),
- an armoured pneumatic hose (4),
- a vacuum cleaner with the blower function and rotational speed control (5),
- two LED lamps with a system (6), and
- a seed container (7).

One of the variable factors checked in the tests was the influence of the speed of air transporting seeds in the pneumatic conduit, i.e., 15, 20, and 25 m/s based on the air speeds used in pneumatic seed drills. Pneumatic conduits in seed drills are arranged in different configurations and they differ in length. For this reason, an S-shaped arrangement of the conduit was chosen, because this configuration is the most common in seed drills with two-meter conduits.

The seed dispenser was made from a typical sowing roller with a diameter of 80 mm (the pin sowing unit), which was driven by a 500 W electric motor. Changes in the voltage resulted in changes to the rotational speed of the sowing roller. As how big the seed mass stream needed to be was known, it was possible to calibrate the dispenser at the appropriate value of rotational speed so as to obtain a sowing density of 400 pcs/m^2^.

The last stage of the experiments involved attempts to set the appropriate speed of air in the pneumatic conduit. The aim of the project was to generate an airstream, transporting seeds in the pneumatic conduit by means of a STANLEY SXVC20PTE vacuum cleaner of Annovi Reverberi S.p.A., Bangkok, Thailand with a nominal power of 1200 W, with the blower function. The power of the vacuum cleaner was adjusted with a potentiometer, which controlled the speed of air within a range of 5–50 m/s.

In order to verify at which speed of transporting air the largest number of small or damaged seeds could be seen, speeds of 15, 20 and 25 m/s were selected. The obtained values were checked with a VOLTCRAFT VPT—100 pressure anemometer with a measuring range of 1–80 m/s and accuracy of +/− 2.5%. 

The following factors were assumed in laboratory tests:

(a) constant factors:
  - triticale seed sowing rate 266.7 g/min, which corresponds to 168 kg/ha,
  - sowing speed of 8 km/h, and
  - cable configuration: K1 (see Figure 2),
(b) variable factors:
  - airstream speed: 15 m/s, 20 m/s, 25 m/s, and
  - type of seeds: select, screenings, waste,
(c) outcome factors:
  - speed of caryopses (m/s), and
  - acceleration of caryopses m/s^2^.

### 2.3. Preparation of Material for Tests with Camera

The graphical data of triticale seeds with different variability (according to the speed and type of seeds) were obtained with a Chronos 1.4 camera of Kron Technologies Inc. Eastlake Dr, Burnaby, Canada, characterized by a high range of image display frequency during the movement of triticale seeds. This is a high-speed camera with a built-in 1.3-megapixel image intensifier, which records transmission rates up to 1057 kbps at a resolution of 1280 × 1024 or even up to 38,500 kbps at lower resolutions.

As a result of the experiment, nine videos were recorded in the MP4 format at a data transmission rate of about 9400 kbps and image resolution of 640 × 360. Frame pacing is important when video devices are used. The more frames per second displayed, the smoother the image is. According to the European TV standard, the correct frame rate is 24 fps. In our tests, the same image display parameters were maintained, i.e., 58 fps, to ensure the repeatability of the results and comparison of speed.

### 2.4. Edge Detection with the Kirsch Mask 

Edge detection is a method of computer image analysis, which includes: image exposure, processing, analysis and classification. The analysis of the resulting image is interpreted by means of: segmentation, location of objects and extraction of the following traits from the image: geometric parameters, color models and image texture. The Kirsch mask consists of pattern recognition by means of segmentation to detect edge pixels in the image. Edge pixels are defined as a set of points (pixels) located on a curve that separates the neighboring points (pixels) or points (pixels) on the other side of the curve, which differ in brightness. The aim of the project was to detect local discontinuities in the brightness level and the outline of the research material [45,46,47]. The Kirsch mask consists of matching the perfect pattern to the edge pixels of the research material. The Kirsch mask in this study used a 5 × 5 matrix. It was based on the free AForge.NET framework (http://www.aforgenet.com/framework/). The authors of this study prepared original functions written in the C# language to import the library to their original ‘PID system’ program. The Kirsch mask edge detection process involved the use of greyscale functions in the ‘PID system’ program to convert 24-bit color images into monochrome (8-bit) greyscale images. In the next step, the Aforge.NET library and original auxiliary functions were used to convert the monochrome images into photos with the Kirsch mask.

### 2.5. Image Processing

At the next stage of the research the training sets of triticale seed classes were designed. In order to obtain digital images, image frames were extracted by means of the VLC Media Player. This free software for watching videos also saves images in .tiff files. As a result, a series of digital photos with a resolution of 640 × 360 was obtained. Table 1 shows the number of photos obtained for each video, allowing for a type of triticale seeds. The photos extracted from the video required preparation of an image pattern in order to determine the representative traits of the image. The original ‘PID system’ (Przybył Image Detector system) software [48] was used for image processing and analysis. It accelerated the generation of the image pattern. The software included a batch module for collective image processing. A series of graphic objects with a resolution of 640 × 360 was imported to the ‘PID system’. The images were collectively filtered with the Kirsch mask and were then exported to the catalogue. At the next stage, the size of the processed image was reduced/cropped with the Kirsch mask in order to obtain a lower resolution image. Like the Kirsch mask, the image processing module in the program allows for collective cropping, having specified the parameters, i.e., image coordinates. This procedure resulted in a set of bitmaps with a resolution of 320 × 280 and 24-bit image depth. Texture descriptors were extracted from the resulting images. The term texture refers to the three-dimensional graphics technique, which is used to present details of the surface of spatial objects by means of bitmap images (textures) or mathematical functions (procedural textures). However, texture cannot always be described in a deterministic way. Statistical methods describe texture as a nondeterministic distribution of dependencies between neighboring pixels. The authors of this study used the Grey-Level Co-occurrence Matrix (GLCM) [49,50] to extract texture descriptors. This statistical method examines texture on the basis of the spatial relationship between pixels. The measurements and statistics of selected texture descriptors were based on the GLCM, which used the individual formulas shown below [51,52,53,54]:

Angular Second Moment (ASM):

(2) $$\mathrm{ASM}=\sum_{i}\sum_{j}{\left\{p\left(i,j\right)\right\}}^{2},$$

Entropy of texture descriptors based on the GLCM:

(3) $$\mathrm{entropy}\;=\;-\sum_{i}\sum_{j}p\left(i,j\right)\log\left(p\left(i,j\right)\right),$$

The correlation that measures the probability of joint occurrence of certain pairs of pixels is calculated in MATLAB with greycoprops:

(4) $$\mathrm{correlation}=\frac{\sum_{i}\sum_{j}\left(\mathrm{ij}\right)p\left(i,j\right)-{µ}_{x}{µ}_{y}}{\sigma_{x}\sigma_{y}},$$

Contrast—local variations in the grey level co-occurrence matrix. This value is calculated in MATLAB by means of the greycoprops function (Park et al., 2001):

(5) $$\mathrm{contrast}=\sum_{i}\sum_{j}p{\left(i-j\right)}^{2}p\left(i,j\right),$$

Inverse Difference Moment—pairs of pixels of feature for texture based on the GLCM:

(6) $$\mathrm{inverse}\;\mathrm{difference}\;\mathrm{moment}\;=\sum_{i}\sum_{j}\frac{1}{1+\;{\left(i-j\right)}^{2}}p\left(i,j\right),$$

where:

μ_x_ and μ_y_—average as a function of the probability of occurrence of pairs of pixels,
σ_x_ and σ_y_—mean standard deviations as a function of the probability of occurrence of similarity between pixels,
p(i, j)—i and j are coordinates of Grey-Level Co-occurrence Matrix.

### 2.6. Construction of the Training Sets

**Figure 3:**
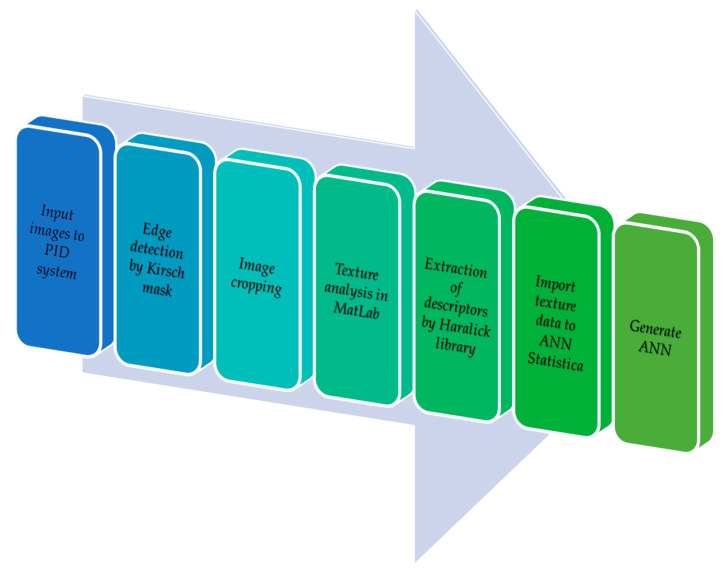
An image recognition algorithm.

When creating artificial neural networks, the first stage involves designing training sets. It is very important to select quality discriminants, i.e., textural descriptors, and the number of training cases (repetitions) to obtain adequate results (Table 1). The texture descriptors data were extracted from the database of the adapted resulting images (i.e., the creation of the image pattern shown in Figure 1), which refer to the identification of contaminants in seeds. The original ‘PID system’ software was used at this stage of image processing (Figure 3) [48]. The descriptors were extracted into MS Excel spreadsheets and four training sets created. The number of their repetitions is shown in Table 1 (Z1, Z2, Z3, and Z4). Each training case included the following texture descriptors [26,51]: angular second moment, entropy, contrast, correlation, and inverse difference. The numerical data (descriptors) were obtained by means of the Matlab and ‘PID system’ software. Next, the training sets were imported into Statistica. Each training set represented a set of the aforementioned descriptors and was divided into training, validation and test subsets at a 2:1:1 ratio [30]. The subsets were not used in the creation of artificial neural networks. Figure 3 shows the image processing, analysis and classification algorithm (based on the interpretation of texture descriptors extracted from the image) in the artificial neural networks creation process.

## 3. Results and Discussion

### 3.1. Results of Artificial Networks

As a result of neural networks training, the networks with the lowest Root Mean Square (RMS) error were obtained (Table 2) [37,55]. In view of the fact that the use of multiple architectures resulted in such high diversity of neural networks, it is very important to choose the ones that will be the most helpful to solve the problem. It might be a good idea to look at the problem area and then select several networks for training and testing. The networks with the smallest error will be the closest to reality and they can be used to solve the problem. Table 2 shows the networks characterized by the highest classification ability for each set. The lowest RMS error, i.e., 0.052 for the training, test and validation sets was noted for the MLP network with five input variables (five texture descriptors) belonging to the input layer, 23 neurons in the hidden layer and three neurons in the output layer. The output layer of the network defined a three-state variable, i.e., types/classes of triticale seeds. The MLP 5-23-3 network was selected from set Z1, for which the resulting image was obtained at a speed of 15 m/s. The MLP 5-6-3 network was selected from set Z2. It had a structure with the same parameters in the input and output layer and six neurons in the hidden layer. The RMS error of the MLP 5-6-3 network was 0.097 at 20 m/s. When the speed was 25 m/s, the MLP 5-3-3 network had a structure with five neurons in the input layer, three neurons in the hidden layer and three neurons in the output layer. The RMS error was 0.187. As the speed of the air transporting triticale seeds increased, so did the error, whereas the classification efficiency decreased. The process of recognition of individual sets was confirmed by the network simulation process with randomly selected outcome images obtained at all speeds of the airstream transporting seeds through the pneumatic channel. As a result, an MLP 5-35-3 network with five input neurons, 35 hidden neurons and three output neurons in the structure was obtained. The RMS error of this network was 0.232.

The correctness of the classification in the test set did not exceed the results in the training set. This means that the networks in Table 2 were not overtrained. In practice, the selected networks were characterized by an excellent classification factor, i.e., above 0.91 for the test set. The best result for the test set was 0.99, when networks with the triticale seed flow speeds of 15 and 20 m/s were simulated. There were also very good results in the network with a seed flow speed of 25 m/s.

### 3.2. Results of Statistical Analysis

A correlation analysis was applied to determine which variable in the set was characterized by high dependence [56,57]. As can be seen in Table 3, Table 4 and Table 5, the entropy variable exhibited the strongest correlation. The highest correlation for the entropy variable was obtained when seed transport at a speed of 15 m/s was simulated. The entropy variable was strongly positively correlated (at 0.92) with the contrast variable and strongly negatively correlated with all the other variables. An increase in the speed of seed transport through the pneumatic channel reduced the strong dependence of the entropy variable in relation to the other parameters. The entropy variable improved the identification of triticale seed classes. It was one of the GLCM texture variables that improved the reproduction of image details. Like other reference publications, our study also proved that entropy had the greatest influence on improvement of the image pattern and texture analysis [58,59]. 

**Figure 4:**
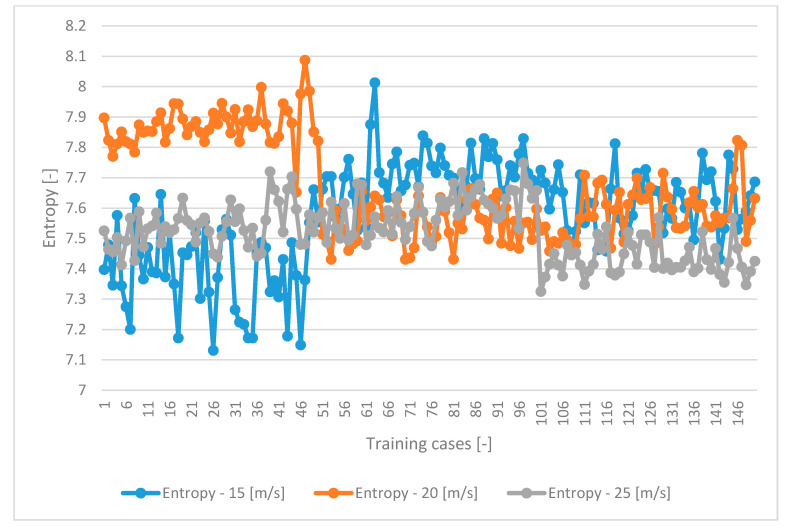
The distribution values of the entropy variable in the dataset depending on the sowing speed.

Figure 4 shows that, due to the change in speed, the entropy variable exhibited linear dependence of data at 15 m/s. Thus, as the speed increased, the frame pacing deteriorated the image quality, which resulted in a non-linear dependence of the entropy variable. Consequently, the correlation value decreased (Table 4 and Table 5). 

By comparison, principal component analysis was applied. Table 6, Table 7 and Table 8 show the eigenvalues of the correlation matrices as measures of variability of the original data presented in the coordinates of the principal components. The specific values of these variances are shown in the second row of the table. The eigenvectors define the individual principal components PC1–PC6. The set of the values of the texture traits at a seed sowing speed of 15 m/s shows the eigenvalue of 5.144 for the first component. The percentage of the variance explained by this component was 85.734%. The second component explained much less variance, i.e., 5.4%, and its eigenvalue was 0.324. According to the Kaiser criterion, the principal component for which the eigenvalue is greater than one is sufficient for interpretation. The first two components carried over 90% of the variance of the original data.

The interdependencies of the variables were compared at a seed sowing speed of 20 m/s. The eigenvalue for the first component was 3.410 and the percentage of the variance it explained was 6.195%. The second component explained much less variance, i.e., 29.739%, and its eigenvalue was 1.487. The first two components carried over 97% of the variance of the original data.

The variance of the values at a seed sowing speed of 25 m/s gave the eigenvalue of 3.274 for the first component. The percentage of the variance explained by this component was 65.485%. The second component explained the variance at 27.133% and its eigenvalue was 1.357. As a result, the first two components carried over 92% of the variance of the original data.

The eigenvalues of the correlation matrices showed that the statistical variance of the data in two dimensions was very good, i.e., approximately over 90%. A change in the seed transport speed decreased the variance of the original data. This was also manifested by weaker correlation of the entropy value with the other variables. 

As a result of the progress made in the research area concentrating on classification issues [26,30,40] with Artificial Neural Networks (ANN, quality changes were observed in relation to farm and food products, which are dictated by consumer expectations [26,34]. This method is based on neural models supported by computer analysis of images, which is faster and more efficient in comparison to the traditional calculating methods. Artificial Neural Networks (ANN) allows us to make more effective simulations of complex processes, among other things, via the process of learning parallel with information processing [29,34]. On the basis of the research results, it was found that the image analysis is an effective technique of recognizing contamination of triticale seeds. Application of traditional methods of classification is slow and often results in improper classification or comes from harvests from previous years. Currently, there is no system that could indicate that a given seed material is contaminated with chaff, which would entail the use of bigger dosage of seed material to ensure the required density of sowing per hectare [60,61]. It sometimes happens that seeds are really small (tailings), especially during dry years. In such a situation, one should decrease seed material dosage in order to ensure a proper sowing. Sowing units used in standard seeders fail to dispense seeds precisely, but only dispense seeds in volume portions. That is why the first step was taken to devise the system of contamination recognition of tiny seeds (tailings). In precise farming, automated seeders are used more and more often and the above system could be applied in those devices [62,63]. It was also noted that high effectiveness in the process of classifying various varieties of grains can be achieved when one takes into consideration proper determination of physical features of grains such as: the mass of a thousand seeds, average geometrical diameter, globosity, grain volume, area, hopper density, real density, porosity and color parameters [64]. It turns out that one can quickly and non invasively evaluate sowing as well as inner structure of grains on the basis of bitmap. The parameters of color can have a tremendous influence on effective evaluation of product quality, because it is responsible for sensory changes in agricultural produce that are subject to research and examination. It can translate into high quality grains, which is the most important indicator in flour production and bread baking [65], cake baking, noodles making and in the brewery industry [27]. The ANN that was created can be a useful tool supporting the existing systems of seeding grains.

## 4. Conclusions

The research showed that the classification error of the generated networks in the recognition of the type of triticale seeds ranged from 6% to 22% in relation to the test set.

The experiment showed that, as the speed increased, the effectiveness of recognition of individual triticale seed classes decreased. The most effective recognition was noted at a speed of 15 m/s. It was the MLP 5-23-3 network, whose RMS error value was 0.052, whereas its classification correctness coefficient was 0.99.

The statistical analysis confirmed the influence of variables on the speed of seed transport. The entropy variable was the most strongly correlated with the other variables, which indicates particular importance of this variable in the dataset.

The entropy variable was the most potent texture factor because it affected image recognition (bitmap) to the greatest extent.

The topology of the MLP network proved to be the best at recognizing the triticale seed classes.

## Figures and Tables

**Table 1:** The distribution of the frequency of training cases depending on the sowing speed and the type of triticale seeds.

Name	Speed [m/s]	Select	Screenings	Chaff	Number of Training Cases
Z1	15	200	200	200	600
Z2	20	50	50	50	150
Z3	25	100	100	100	300
Z4	15, 20, 25	200, 200, 200	200, 200, 200	200, 200, 200	1800

**Table 2:** Network training results.

Name of Learning Set	Z1	Z2	Z3	Z4
Ann model	MLP 5-23-3	MLP 5-6-3	MLP 5-3-3	MLP 5-35-3
Training error	0.060	0.095	0.160	0.226
Validation error	0.038	0.091	0.186	0.251
Testing error	0.058	0.103	0.214	0.220
Quality of learning	0.990	0.973	0.933	0.894
Quality of validation	0.999	0.999	0.947	0.844
Quality of testing	0.993	0.990	0.920	0.913
Training cases	600	150	300	1800
Training algorithm	BP50, CG257b	BP50, CG227b	BP50, CG69b	BP50, CG1094b

**Table 3:** The correlation matrix of set Z1.

	Angular Second Moment	Contrast	Correlation	Inverse Difference Moment	Entropy
Angular Second Moment	1				
Contrast	−0.851	1			
Correlation	0.779	−0.830	1		
Inverse Difference Moment	0.900	−0.780	0.788	1	
Entropy	−0.935	0.925	−0.859	−0.927	1

**Table 4:** The correlation matrix of set Z2.

	Angular Second Moment	Contrast	Correlation	Inverse Difference Moment	Entropy
Angular Second Moment	1				
Contrast	−0.526	1			
Correlation	0.046	−0.778	1		
Inverse Difference Moment	0.989	−0.438	−0.061	1	
Entropy	−0.967	0.696	−0.275	−0.932	1

**Table 5:** The correlation matrix of set Z3.

	Angular Second Moment	Contrast	Correlation	Inverse Difference Moment	Entropy
Angular Second Moment	1				
Contrast	−0.504	1			
Correlation	0.084	−0.604	1		
Inverse Difference Moment	0.954	−0.350	−0.041	1	
Entropy	−0.907	0.772	−0.361	−0.786	1

**Table 6:** The eigenvalues of the correlation matrix (15 m/s).

Eigenvalues	PC1	PC2	PC3	PC4	PC5	PC6
Variance	5.144	0.324	0.273	0.180	0.060	0.019
% of var.	85.734	5.400	4.546	2.995	1.008	0.316
Cumulative% of var.	85.734	91.134	95.680	98.676	99.694	100

**Table 7:** The eigenvalues of the correlation matrix (20 m/s).

Eigenvalues	PC1	PC2	PC3	PC4	PC5
Variance	3.410	1.487	0.090	0.010	0.004
% of var.	68.195	29.739	1.797	0.190	0.078
Cumulative% of var.	68.195	97.935	99.732	99.922	100.00

**Table 8:** The eigenvalues of the correlation matrix (25 m/s).

Eigenvalues	PC1	PC2	PC3	PC4	PC5
Variance	3.274	1.357	0.296	0.062	0.011
% of var.	65.484	27.133	5.916	1.242	0.224
Cumulative% of var.	65.484	92.618	98.534	99.776	100

## Data Availability

Not applicable.
